# Chromosome Missegregation Associated with RUVBL1 Deficiency

**DOI:** 10.1371/journal.pone.0133576

**Published:** 2015-07-22

**Authors:** Christian Gentili, Dennis Castor, Svenja Kaden, David Lauterbach, Mario Gysi, Patrick Steigemann, Daniel W. Gerlich, Josef Jiricny, Stefano Ferrari

**Affiliations:** 1 Institute of Molecular Cancer Research of the University of Zurich and the ETH Zurich, Winterthurerstrasse 190, CH-8057, Zurich, Switzerland; 2 Institute of Biochemistry, Schafmattstrasse 18, HPM E17.2, Swiss Institute of Technology Zurich (ETHZ), CH-8093, Zurich, Switzerland; Institut de Génétique et Développement de Rennes, FRANCE

## Abstract

RUVBL1 (RuvB-like1) and RUVBL2 (RuvB-like 2) are integral components of multisubunit protein complexes involved in processes ranging from cellular metabolism, transcription and chromatin remodeling to DNA repair. Here, we show that although RUVBL1 and RUVBL2 are known to form heterodimeric complexes in which they stabilize each other, the subunits separate during cytokinesis. In anaphase-to-telophase transition, RUVBL1 localizes to structures of the mitotic spindle apparatus, where it partially co-localizes with polo-like kinase 1 (PLK1). The ability of PLK1 to phosphorylate RUVBL1—but not RUVBL2—*in vitro* and their physical association *in vivo* suggest that this kinase differentially regulates the function of the RuvB-like proteins during mitosis. We further show that siRNA-mediated knock-down of RuvB-like proteins causes severe defects in chromosome alignment and segregation. In addition, we show that the ATPase activity of RUVBL1 is indispensable for cell proliferation. Our data thus demonstrate that RUVBL1 is essential for efficient mitosis and proliferation.

## Introduction

Genomic instability, ranging from loss of heterozygosity, gene amplifications, chromatid breaks and chromosomal rearrangements to the loss or gain of entire chromosomes, is one of the key characteristics of cancer cells. The molecular transactions underlying the above aberrations have not been fully elucidated, but a subset of these events can be ascribed to the malfunction of DNA helicases. Bloom Syndrome, Werner Syndrome and Rothmund-Thomson Syndrome/ Rapadillino, severe pathologies associated with cancer predisposition, premature ageing and developmental abnormalities, are linked to mutations in genes of the *RecQ* helicase family *BLM*, *WRN* and *RecQ4* genes, respectively [1], and cell lines isolated from patients afflicted with these syndromes display considerable genomic instability. That helicase malfunction might destabilize the genome should come as no surprise, given the key roles played by this important class of enzymes in all pathways of DNA metabolism that involve unwinding of the duplex, such as transcription, replication, recombination and repair [2]. The RecQ family helicases are believed to function primarily during the S- and G_2_-phases of the cell cycle, where they participate in stabilizing replication forks and help resolve recombination intermediates. However, more recent evidence points to a mitotic role for BLM [3] that, together with the DNA translocase PICH [4] and the topoisomerase TOP2A [5], participates in the resolution of ultrafine bridges deriving from incomplete sister chromatid disjunction at anaphase, a critical stage in the cell cycle stage during which chromosome missegregation can give rise to aneuploidy [6].

Analysis of the human mismatch repairosome [7] identified RuvB-like 1 (RUVBL1, also known as Pontin, RVB1, Tip49a, ECP-54, Tih1, p50 and Tap54β) and RuvB-like 2 (RUVBL2, also known as Reptin, RVB2, Tip49b, ECP-51, Tih2, p47 and Tap54α). These polypeptides belong to the AAA+ (ATPases associated with various cellular activities) superfamily and have been proposed to possess helicase activity, though their ability to unwind DNA is still subject to debate [8–11]. These genes are essential in both yeast [9,12] and mice [13] and are upregulated in cancer [14,15].

RUVBL1/2 are part of large multiprotein complexes such as NuA4 and INO80 [16–18] and they were shown to regulate the abundance of the *Fanconi anemia* core complex [13], which implicates them in DNA damage response. They form a heterodimer [7,19] that assembles into a 650 kDa molecular machine formed by interaction of two hexamers of heterodimers [10,11,18,20,21]. Functionally, the RUVBL1/2 complex was shown to play a role in chromatin remodeling and transcription (for reviews see [22,23]) and to interact with the phosphatidylinositol kinase-like kinases (PIKKs) ATM, ATR and DNA-PK [24] in DNA damage signaling.

Interestingly, RUVBL1/2 are mostly nuclear in interphase and undergo relocalization in mitosis; RUVBL2 was found to localize to the central spindle and the midbody [25,26], while RUVBL1 was shown to be present at centrosomes and the mitotic spindle [27,28]. Based on the RUVBL1 interaction with γ-tubulin and on defects in microtubule polymerization upon its depletion in *Xenopus laevis* egg extracts, RUVBL1 was proposed to function in microtubule assembly [29]. A role for RUVBL1/2 as chromatin decondensation factors at the end of mitosis was recently described [30].

In an attempt to elucidate the possible link of the RUVBL1/2 proteins to DNA metabolism, we examined their localization by indirect immunofluorescence. In the course of this investigation, we noticed that the polypeptides underwent dramatic relocalization during the cell cycle. Most notably, the RUVBL1/2 heterodimer appeared to dissociate during late telophase and the signal of RUVBL1 co-localized with that of polo-like kinase 1 (PLK1) in the interphase bridge. The latter observation was underscored by the finding that RUVBL1 associates with PLK1 during mitosis and that it is phosphorylated by this kinase *in vitro* on threonine 239. RNAi-mediated depletion of RUVBL1 gave rise to severe chromosome misalignment and lagging chromosomes. Furthermore, inducible knock-down of endogenous RUVBL1 and simultaneous expression of an ATPase-dead RUVBL1 mutant impaired cell proliferation. Taken together, our findings demonstrate that RUVBL1 plays an essential role in the maintenance of genomic stability and cell cycle progression.

## Results

### RUVBL1 and RUVBL2 dissociate during mitosis

**Fig 1:**
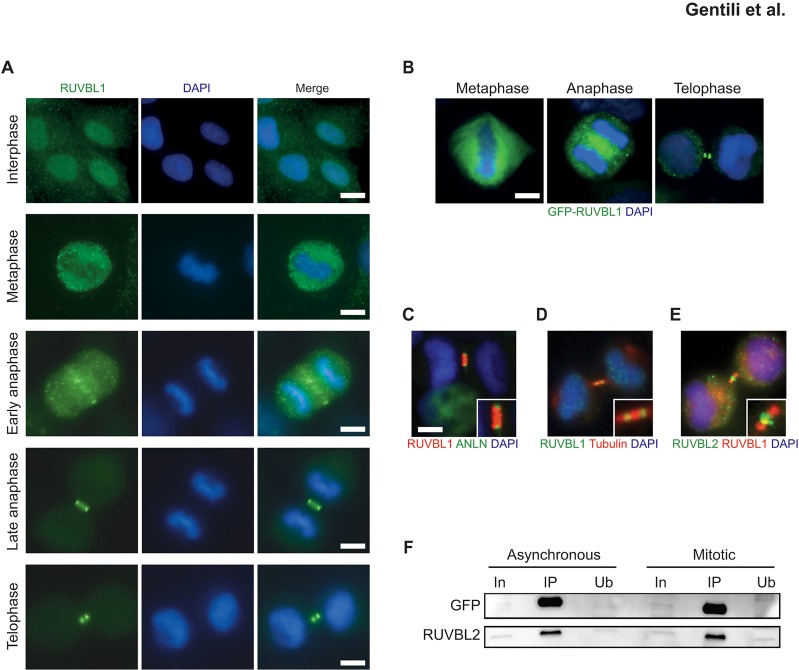
Chromosomal passenger protein-like staining of RUVBL1/2 during cell division. **(A)** Methanol-fixed U2OS cells were stained with anti-RUVBL1 antibody (green) and DAPI (blue) in interphase and various stages of mitosis. At least 50 events were examined (N≥50). **(B)** Localization of GFP-mRuvBL1 at different stages of mitosis in HeLa cells. (N≥50). **(C)** Co-staining of RUVBL1 (red) and GFP-Anillin (green) in dividing cells. DNA is counterstained with DAPI (blue). Scale bar, 5 μm; (N≥50). **(D)** Co-staining of RUVBL1 (green) and β-tubulin (red) in dividing cells. DNA is counterstained with DAPI (blue). (N≥50) **(E)** Co-staining of RUVBL1 (red) and RUVBL2 (green) showing their different localization during late telophase. DNA is counterstained with DAPI (blue). (N≥50). **(F)** Whole cell extracts (1 mg) from asynchronous or double-thymidine/nocodazole-synchronized GFP-mRuvBL1 expressing HeLa cells were used for immunoprecipitation with a GFP-trap antibody. Precipitated material was analyzed with antibodies to GFP and RUVBL2. In: input (2.5%); IP: immunoprecipitated fraction; Ub: unbound fraction.

We used indirect immunofluorescence (IF) to examine the localization of endogenous RUVBL1/2 in U2OS cells (Fig 1A and 1C–1E) and of GFP-tagged human or mouse RUVBL1 (Fig 1B) expressed in HeLa cells from bacmid constructs at similar-to-endogenous protein levels [31]. Anillin was used as marker for the cyokinetic furrow (Fig 1C). We observed that the RUVBL1 signal was diffused throughout the nucleus during interphase, but that its localization underwent dramatic changes during mitosis and cytokinesis. Specifically, RUVBL1 appeared to be largely excluded from metaphase chromosomes, as also reported by others [27,28], while it relocated to the central spindle during the anaphase-to-telophase transition (Fig 1A). Later, RUVBL1 was observed at the sides of the closing cytokinetic furrow (Fig 1A and 1C) and it finally accumulated to two distinct foci in the mature intracellular bridge (Fig 1A and 1B, telophase), where it co-localized with β-tubulin (Fig 1D). Specificity of the RUVBL1 antibody was evident from lack of staining upon pre-incubation of the antibody with purified His-tagged RUVBL1 (S1A Fig), as well as after siRNA-mediated depletion of RUVBL1 (S1B Fig). In addition, the same staining pattern could be observed using an antibody raised in another species against a different epitope of RUVBL1 (S1C Fig).

Interestingly, RUVBL2 did not co-localize with RUVBL1 at this time, but rather remained in the central region of the midbody (Fig 1E). This finding was unexpected and novel, since RUVBL1 and RUVBL2 are known to exist as a dimer of heterohexameric rings [11,18,20,21,32]. The separate localization of RUVBL1 and RUVBL2 at the midbody suggests that the complex dissociates during mitosis and that the two proteins may have distinct roles and/or may be differentially-regulated at this point of the cell cycle.

To biochemically test this hypothesis, we examined GFP-hRUVBL1 HeLa cells that were either grown asynchronously or that were arrested in mitosis by a combined double-thymidine block-release and nocodazole treatment. Immunoprecipitation of GFP-hRUVBL1 revealed an interaction with RUVBL2 under both conditions (Fig 1F). From these data and the results presented above, we conclude that interphase RUVBL1/2 complexes exist throughout the cell cycle, persist until anaphase and disassemble during cytokinesis.

### siRNA-mediated knock-down of RUVBL1 gives rise to lagging chromosomes

**Fig 2:**
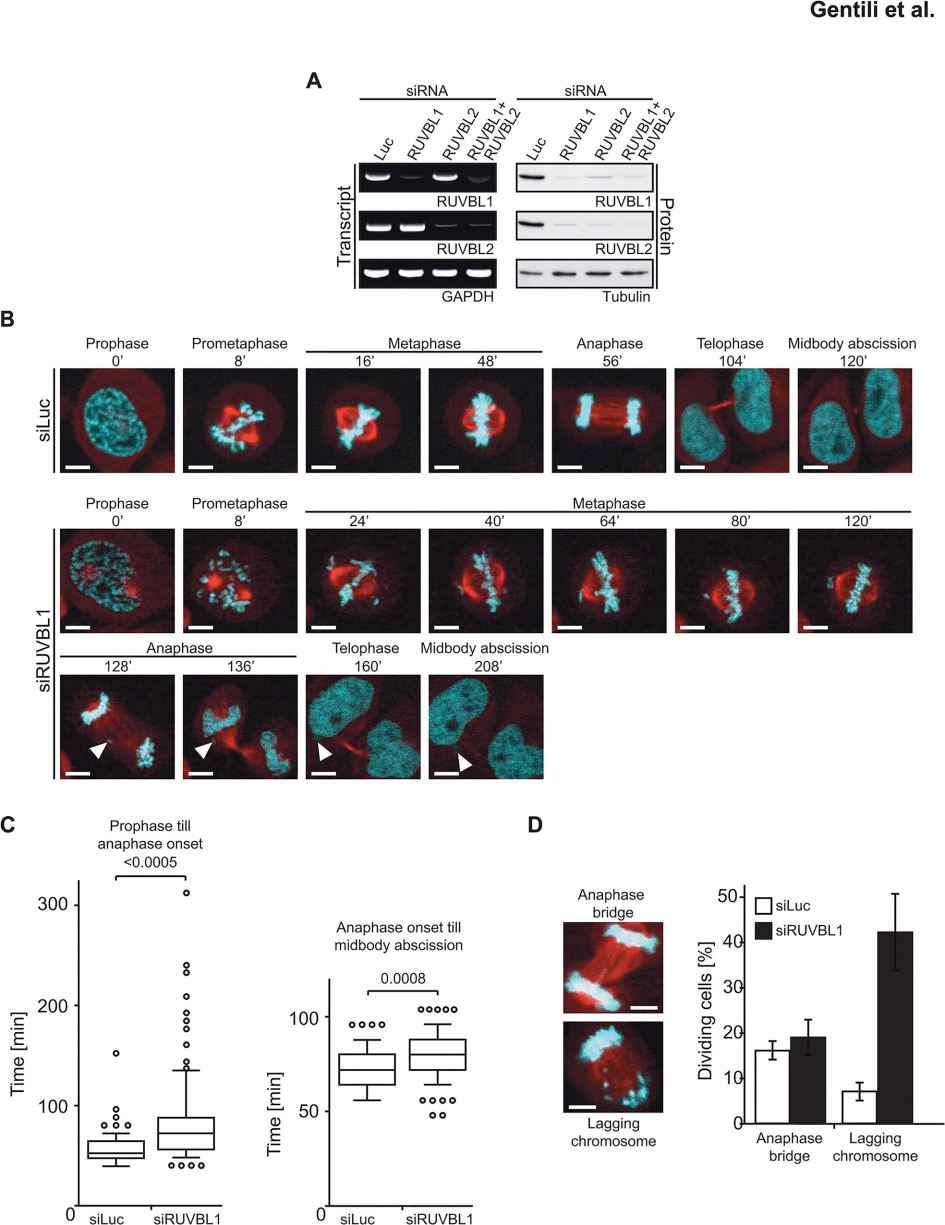
RUVBL1 depletion affects the length of mitosis and results in lagging chromosomes. **(A)** HeLa cells were transfected with the indicated siRNA oligos and analyzed by RT-PCR and immunoblot 48 h post-transfection. **(B)** Confocal live imaging after siRNA-mediated knock-down was performed. The figure shows stills of control or RUVBL1 siRNA-treated cells. Lagging chromosomes are indicated with arrowheads. DNA is shown in cyan and α-tubulin in red. **(C)** Early mitotic progression was analyzed by measuring the time from prophase (nuclear envelope breakdown: NEB) to anaphase onset (left panel). Mitotic exit was estimated by measuring the period of time from anaphase onset until accomplished cytokinesis (right panel). The bottom and top of the boxes represent the first and third quartiles, respectively. Horizontal lines inside the boxes represent the median of the data points (n = 100). Whiskers span the 10th and 90th percentiles, with individual dots showing data points that lie outside of these percentiles. Statistical significance was determined by Mann–Whitney test and *p*-values are indicated above. **(D)** Occurrence of aberrant mitotic phenotypes was quantified by analyzing 100 cell divisions in each cell line.

Given the dissociation of the RUVBL1/2 complex and the re-localization of the proteins to the midbody during cytokinesis (Fig 1), we asked whether the two polypeptides might assume distinct roles during this cell cycle stage. To this end, we knocked down RUVBL1 or RUVBL2 in HeLa cells by siRNA. Interestingly, although the siRNAs were specific for the respective mRNAs, as shown by RT-PCR (Fig 2A, left panel), we observed a simultaneous downregulation of both RUVBL1 and RUVBL2, irrespective of whether siRNA against RUVBL1 or 2 was used (Fig 2A, right panel), as previously reported [24,33,34]. That RUVBL1/2 levels remained constant during mitosis (Fig 1F), and were clearly detectable as separate entities when the two polypeptides did not interact (Fig 1E), confirms previous studies on the stability of pre-existing vs. newly synthesized populations of the two proteins [34] and suggests that RUVBL1/2 might be available for interaction with alternative partners during this cell cycle stage, in a manner that is possibly controlled by post-translational modifications (see below).

To address the effect of protein depletion on mitotic progression, we used HeLa cells stably-expressing the mRFP-tagged histone variant H2B, as well as EGFP-tagged α-tubulin [35]. The cells were transfected with RUVBL1 siRNA, and confocal 3-D time-lapse movies were recorded 48 hours later (Fig 2B). RUVBL1-depleted cells were delayed in the progression from prophase to the onset of anaphase (Fig 2B and 2C, left panel) and showed a large increase in the incidence of lagging chromosomes (Fig 2B and box2D). The number of anaphase bridges, such as those observed upon BLM- or PICH-depletion [36], did not increase in RUVBL1-depleted cells (Fig 2D). Furthermore, the progression from anaphase onset to midbody-associated microtubule disassembly, which occurs at abscission [35], was delayed only to a minor extent (Fig 2C, right panel).

To exclude the possibility that the lagging chromosomes arose through off-target effects of the deployed siRNA, we generated stable U2OS T-REx cell lines harboring doxycycline-inducible constructs encoding either shRNA to endogenous RUVBL1 or to RUVBL1, together with a cDNA expression vector encoding wild type mouse RuvBL1. Mouse RuvBL1 differs from the human polypeptide by a single amino acid, and this difference renders the mouse mRNA resistant to shRNA targeting human RUVBL1. In this cell system, depletion of endogenous RUVBL1 also increased the incidence of lagging chromosomes, whereas concomitant expression of murine RuvBL1 rescued this pathogenic phenotype (S2A and S2B Fig).

### RUVB-like 1 is a substrate for Polo-like Kinase 1 *in vitro*

**Fig 3:**
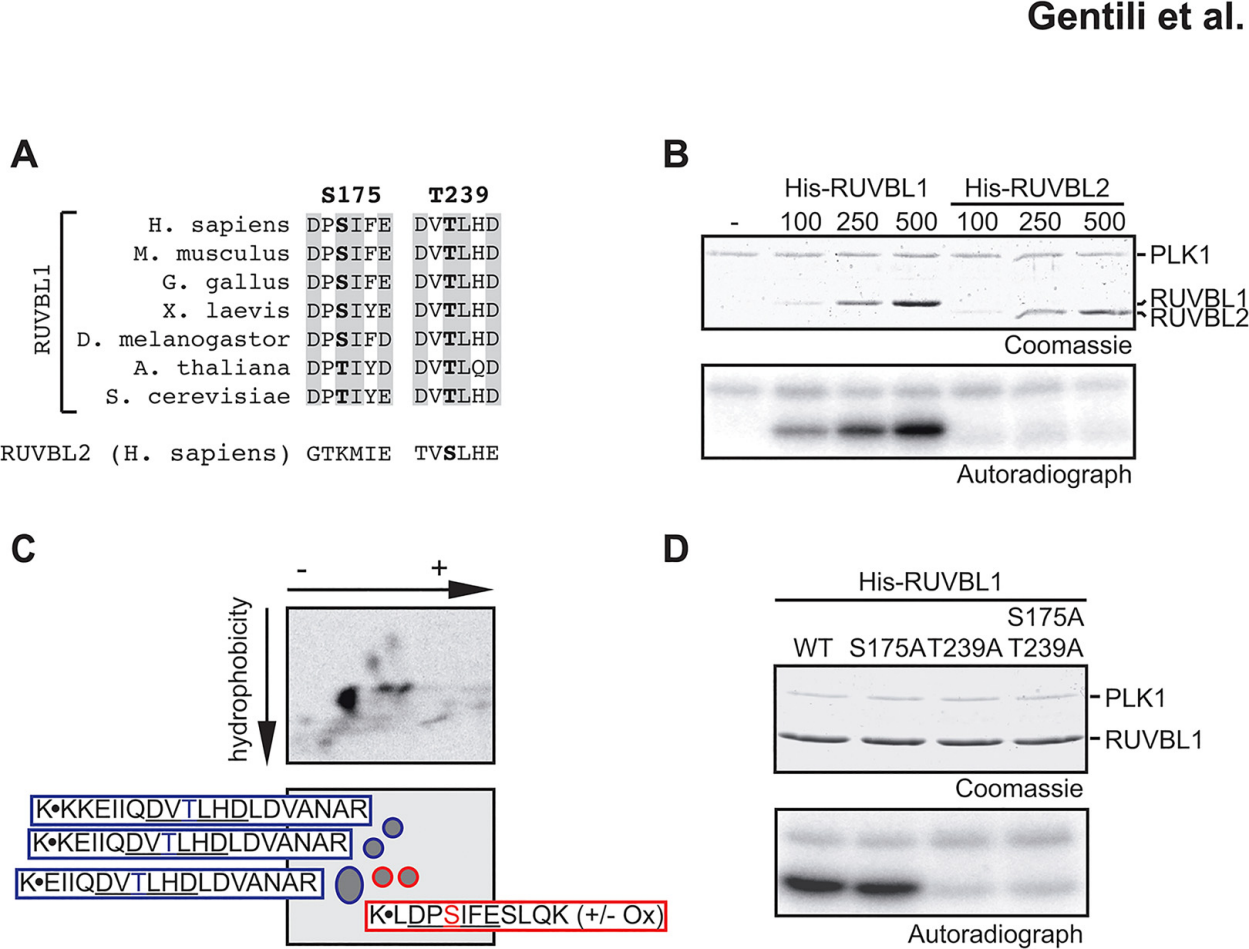
*In vitro* phosphorylation of RUVBL1 by PLK1. **(A)** Protein sequences were obtained from http://www.ncbi.nlm.nih.gov and scanned for PLK1 consensus motifs [37]. Corresponding sequences of human RUVBL2 are shown by comparison below the alignments. Crucial amino acids are shown in gray. **(B)** The indicated amounts of His-tagged RUVBL1 or RUVBL2 were incubated with PLK1 in the presence of [γ-^32^P]ATP and protein phosphorylation was monitored by autoradiography. **(C)** Bands corresponding to phosphorylated RUVBL1 were excised and in-gel digested with trypsin. Eluted peptides were separated in two dimensions by their hydrophobicity and charge. Potential sequences were assigned to the obtained phosphopeptide patterns after autoradiography. Trypsin digestion sites are indicated with K•X. **(D)** Purified His-tagged RUVBL1 mutants were incubated with PLK1 in the presence of [γ-^32^P]ATP and protein phosphorylation was monitored by autoradiography.

The differences in RUVBL1 and RUVBL2 localization (Fig 1), the requirement for RUVBL1 in unperturbed mitosis (Fig 2) and the observation that the RUVBL1 and RUVBL2 signals separate in the midbody suggested that the two polypeptides may assume distinct biological roles at this point of mitosis. This prompted us to study the regulation of RUVBL1 and RUVBL2 during cell division. Given the important roles of protein kinases in mitosis, we searched the amino acid sequences of RUVBL1 and RUVBL2 for consensus phosphorylation motifs of the essential mitotic kinases CDK1, Aurora B or PLK1. We did not identify CDK1 or Aurora B consensus motifs, but RUVBL1 harbored two potential PLK1 consensus sites (D/E-x-**S/T**-Ф-x-D/E, where Ф can be any hydrophobic amino acid, [37]). These sites are not conserved in RUVBL2, although the proteins share a 66% amino acid homology (Fig 3A and S3A Fig). In RUVBL1, the PLK1 motifs lie within a domain of the protein that is not present in the prokaryotic orthologs (S3B and S3C Fig), suggesting that these domains harbor unique functions in eukaryotes [10]. This notion was further strengthened by the fact that S_175_ and T_239_ as well as the PLK1 recognition motifs of RUVBL1 are evolutionarily conserved from yeast to man (Fig 3A). Hence, we wanted to assess whether it is a substrate for this kinase. We therefore established an *in vitro* assay, in which increasing amounts of His-tagged RUVBL1 were incubated with purified recombinant PLK1 in the presence of [γ-^32^P] ATP (Fig 3B and S4A Fig). RUVBL1 was indeed phosphorylated by PLK1, in contrast to RUVBL2 (Fig 3B). To identify the PLK1-modified amino acids, the bands corresponding to phosphorylated RUVBL1 were excised and in-gel digested with trypsin. The extracted peptides were then separated by two-dimensional thin layer electrophoresis/chromatography according to their charge and hydrophobicity. Two distinct peptide patterns were observed, wherein the spots assigned to the peptide containing T_239_ appeared to be phosphorylated to a greater extent than those assigned to S_175_ (Fig 3C). To support our findings, we generated His-tagged RUVBL1 mutants S_175_A and T_239_A, as well as the double mutant S_175_A/T_239_A. These polypeptides were expressed in *E*. *coli* and purified to near homogeneity (S4B Fig). Equal amounts of each mutant were then tested in the *in vitro* PLK1 assay. In contrast to the wild type and the S_175_A proteins, phosphorylation of the T_239_A and S_175_A/T_239_A mutants was substantially diminished (Fig 3D), suggesting that T_239_ of RUVBL1 is the primary phosphorylation site for PLK1 *in vitro*.

Previous studies reported that RUVBL1 and RUVBL2 exist predominantly in a dodecameric complex [11,20,32]. To test whether T_239_ of RUVBL1 is accessible for phosphorylation in the RUVBL1/2 complex, we incubated the purified heterodimer with PLK1 in the presence of [γ-^32^P] ATP. Because RUVBL1 could still be phosphorylated by PLK1 (S4C Fig) we conclude that interaction with RUVBL2 does not hinder phosphorylation of RUVBL1.

Taken together, these results demonstrate that PLK1 phosphorylates RUVBL1 *in vitro* primarily on T_239_ and that this phosphorylation is unaffected by RUVBL1 binding to RUVBL2. This implies that RUVBL1 can be phosphorylated by PLK1 prior to dissociation from RUVBL2.

### RUVBL1 and PLK1 interact during cell division

**Fig 4:**
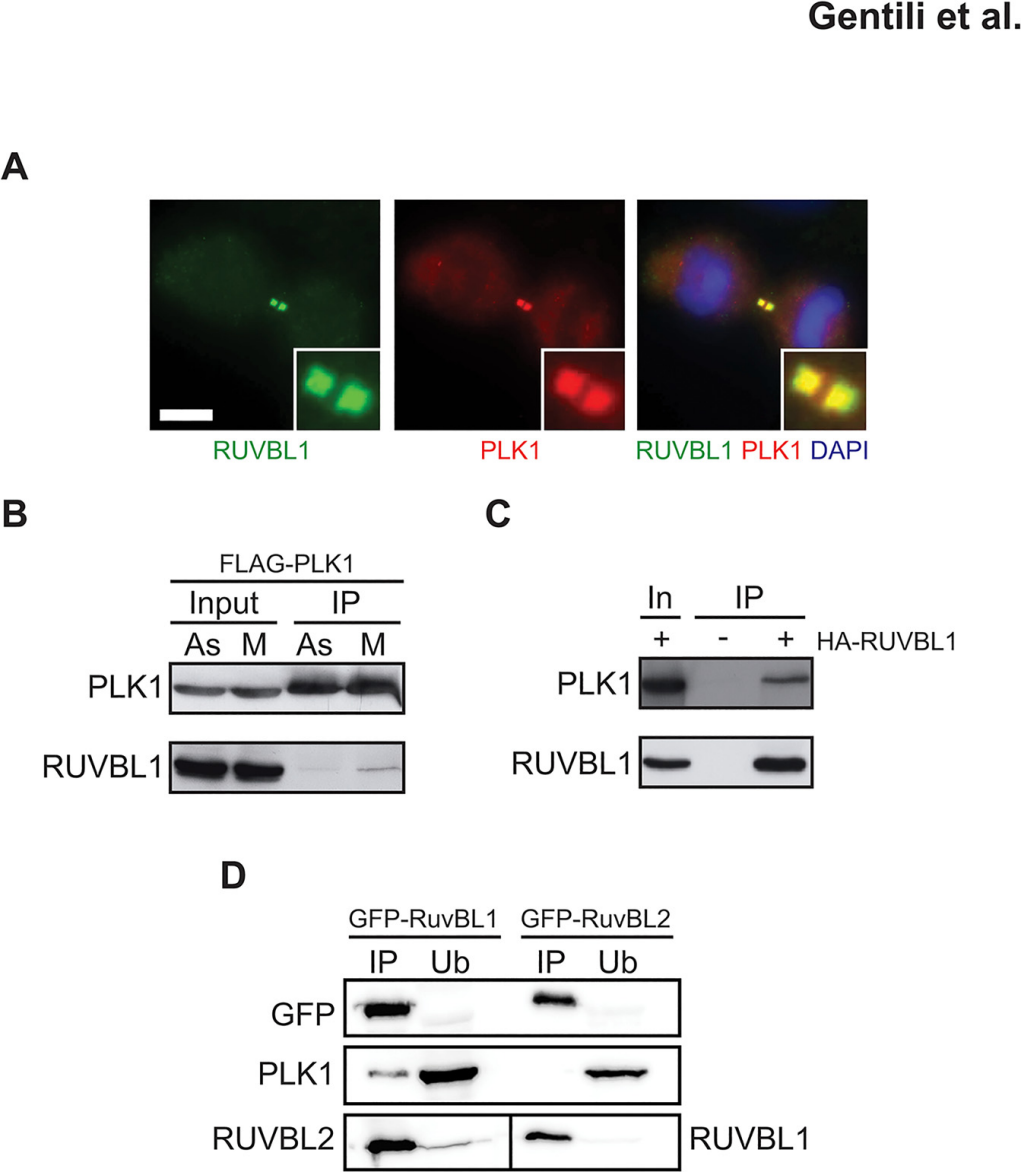
RUVBL1 and PLK1 interact during mitosis. **(A)** Co-staining of RUVBL1 (green) and PLK1 (red) in dividing HeLa cells. DNA is counterstained with DAPI (blue). At least 50 events were examined (N≥50). **(B)** Extracts of asynchronous (As) or nocodazole-arrested (M) FLAG-PLK1-transfected 293T cells were immunoprecipitated with anti-FLAG beads and bound material was analyzed for the presence of RUVBL1. Input (5%, both for As and M); IP: immunoprecipitated fraction. **(C)** Reciprocal immunoprecipitation of nocodazole-arrested 293T cells transfected with HA-tagged RUVBL1. In: input (5%); IP: immunoprecipitated fraction. **(D)** Extracts of double-thymidine/nocodazole-synchronized mouse GFP-RuvBL1 expressing HeLa cells were immunoprecipitated and proteins detected with the indicated antibodies. In: input (2.5%); IP: immunoprecipitated fraction; Ub: unbound fraction.

Considering the established role and localization of PLK1 during mitosis [38] and the fact that RUVBL1 was phosphorylated by PLK1 *in vitro* (Fig 3), we asked whether the two proteins interact *in vivo*. To this end, we examined mitotic HeLa cells by indirect immunofluorescence and observed that the signals of RUVBL1 and PLK1 at the two distinct foci in the mature intracellular bridge coincided (Fig 4A). Using an independent approach, we transiently expressed FLAG-tagged PLK1 in 293T cells, synchronized cells with nocodazole and performed an immunoprecipitation with an anti-FLAG antibody. RUVBL1 was selectively enriched in the immunoprecipitate from mitotic extracts as compared to extracts of an asynchronous cell population (Fig 4B). To further substantiate this evidence, we performed a reciprocal immunoprecipitation experiment with HA-tagged RUVBL1 and noted an enrichment of PLK1 in extracts of cells arrested in mitosis (Fig 4C). Furthermore, we examined HeLa cells stably expressing either GFP-mRuvBL1 or GFP-mRuvBL2. Immunoprecipitation of the tagged proteins followed by Western blot analysis revealed that whereas mRuvBL1 and mRuvBL2 were always detected as a complex, PLK1 selectively interacted with GFP-mRuvBL1 (Fig 4D). Taken together, the above evidence suggests that PLK1, an established regulator of midbody formation [39], phosphorylates RUVBL1 *in vivo*.

### RUVBL1 ATPase activity is indispensable for cell proliferation

**Fig 5:**
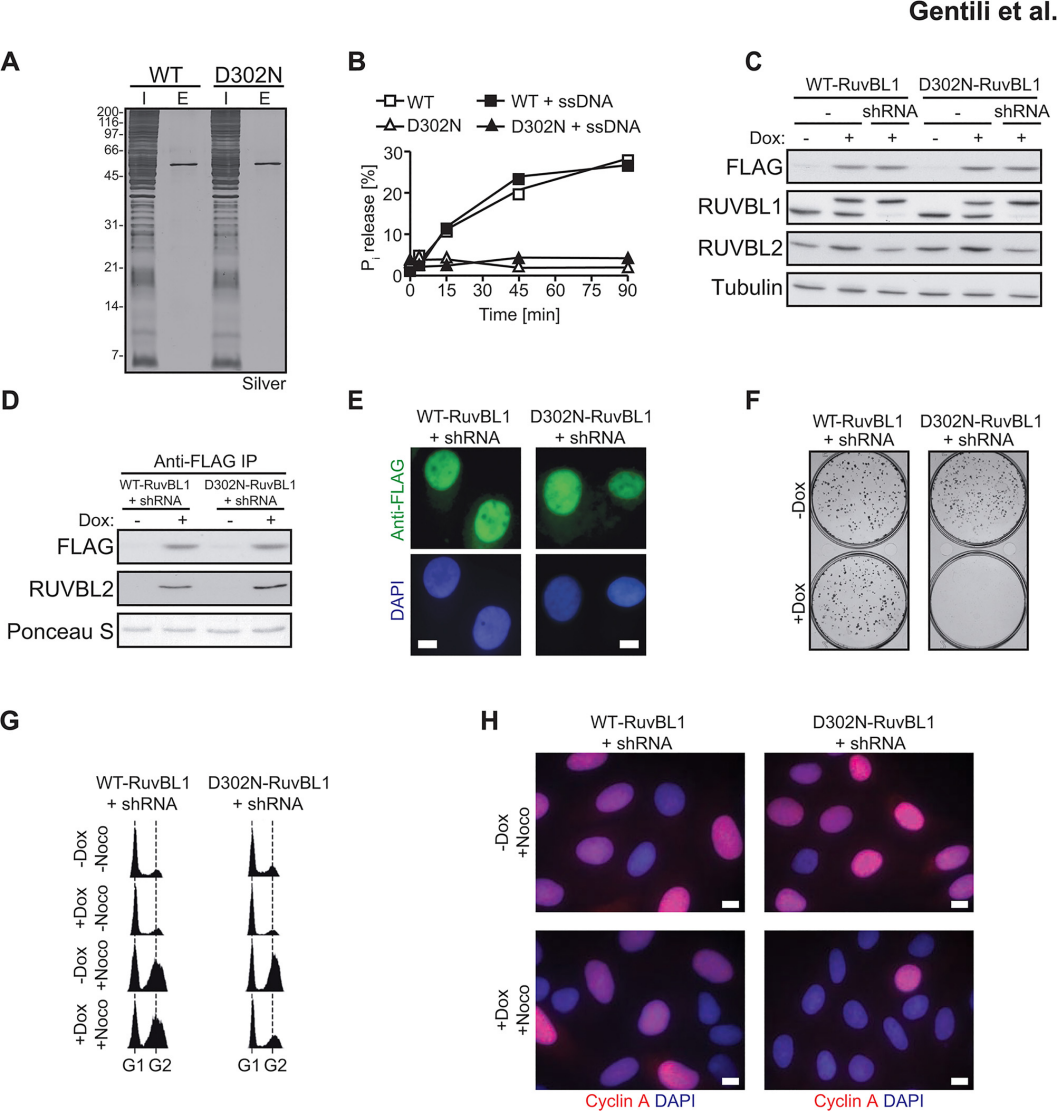
ATPase activity of mammalian RUVBL1 is indispensable for cell proliferation. **(A)** FLAG-tagged RUVBL1 was transiently expressed in 293T cells and protein extracts were prepared 48 h after transfection. Anti-FLAG beads were used to isolate the tagged RUVBL1. Silver staining PAGE shows the purity of the isolated material. **(B)** ATPase activity was measured by incubating purified FLAG-tagged RUVBL1 with [γ-^32^P]ATP for the times indicated, either in the presence or absence of ssDNA. **(C)** U2OS T-REx cells carrying stably-integrated, inducible shRNA-resistant murine RuvBL1 variants, were treated with doxycycline for 96 h (lane 2 and 5). In addition, the cell lines were stably-transfected with an inducible shRNA construct that enables downregulation of endogenous RUVBL1 (lane 3 and 6) after doxcycycline addition. Parental cells are shown for comparison (lanes 1 and 4). **(D)** Anti-FLAG immunoprecipitates confirm the interaction of exogenous RuvBL1 with endogenous RUVBL2. **(E)** Paraformaldehyde-fixed cells were stained with anti-FLAG antibody (green) and DAPI (blue) after 96 h of doxycycline induction. Scale bar, 5 μm. **(F)** Clonogenic survival assay after doxycycline-induced expression of the wild type or D_302_N RuvBL1 variants. The experiments were performed in triplicates and a representative image is shown (quantification of the assay is shown in S5 Fig). **(G)** Cells were induced with doxycycline or left untreated for 96 hours. To arrest the cells in mitosis, nocodazole was added for additional 16 h. DNA content was measured by flow cytometric analysis of propidium iodide-stained cells. **(H)** Paraformaldehyde-fixed cells were stained with anti-Cyclin A antibody (red) and DAPI (blue) after 96 h of doxycycline induction and 16 h of nocodazole treatment. Scale bar, 5 μm.

The ATPase and helicase activities of RuvB-like proteins are still subject to debate, most likely due to differences in protein expression and purification protocols, or due to the fact that the proteins were expressed in heterologous systems [8–11,16]. Hence, to learn whether the ATPase activity of RUVBL1 is essential for its biological function and to avoid issues encountered in other laboratories, we set out to express the protein in human cells. FLAG-tagged RUVBL1 was transiently over-expressed in 293T cells and purified by affinity chromatography on anti-FLAG beads under stringent conditions. After this enrichment, only a single band was seen on a silver-stained polyacrylamide gel (Fig 5A), indicating that the amount of contaminating polypeptides was negligible. Purified wild type FLAG-RUVBL1 displayed robust ATPase activity. To ensure that the enzymatic activity measured in our assay was due to RUVBL1 and not to residual amounts of the endogenous RUVBL1 or the RUVBL1/2 complex, we expressed a mutant carrying a D_302_N mutation in the putative ATPase active site of RUVBL1 as control (Fig 5B) [10,40]. Having confirmed the absence of ATPase activity in the D_302_N FLAG-RUVBL1 mutant preparation, we set out to generate stable U2OS T-REx cell lines harboring wild type or D_302_N doxycycline-inducible RuvBL1 constructs (Fig 5C, lanes 2 and 5). We chose to express the murine RuvBL1 protein, which differs from the human polypeptide by only a single amino acid (isoleucine instead of leucine). As mentioned above, this difference allowed us to express in the U2OS T-REx cells also a doxycycline-inducible shRNA targeting the endogenous RUVBL1 protein (Fig 5C, lanes 3 and 6). To confirm the integrity of our system, we isolated the FLAG-tagged proteins from the cell lines after four days of doxycycline treatment and observed similar levels of co-immunoprecipitated RUVBL2 (Fig 5D). In addition, both the wild type and the D_302_N RuvBL1 mutant protein accumulated in the nucleus (Fig 5E). To assess the long-term consequences of the lack of RUVBL1 ATPase activity, we performed clonogenic survival assays. In the uninduced state, cells expressing the wild type or the D_302_N variant formed colonies of similar size and number. After doxycycline addition, the colonies of cells expressing wild type RuvBL1 were similar to those seen in the uninduced state. In contrast, expression of the D_302_N mutant completely suppressed colony formation (Fig 5F and S5 Fig) demonstrating that the ATPase activity of RUVBL1 is essential for cell proliferation. This agrees with findings that RUVBL1 knock-out mice are embryonic lethal and that a conditional knock-out in hematopoietic cells results in bone marrow failure [41]. Our data strongly suggest that the observed toxicity is not due to loss of the polypeptide, but rather to the loss of its ATPase activity.

In an attempt to understand the colony growth defect in the U2OS cells expressing RuvBL1 D_302_N, we analyzed the cells by flow cytometric analysis. The cell cycle profiles of uninduced- and doxycycline-induced cells looked very similar, with most cells in G_1_. However, when the cells were treated with nocodazole, only cells expressing wild type RuvBL1 displayed an increase in the G_2_/M fraction, which was indicative of mitotic arrest (Fig 5G). This suggested that cells expressing the D_302_N mutant either remained in G_1_, or that their spindle assembly checkpoint was defective. To further characterize the cell cycle stage of D_302_N expressing cells, we stained by immunofluorescence for cyclin A—a marker of the S/G_2_ phase of the cell cycle. In agreement with the flow cytometric results, the majority of D_302_N RuvBL1-expressing cells were cyclin A-negative after nocodazole challenge, compared to the wild type RuvBL1-expressing cells (Fig 5H).

## Discussion

The RUVBL1 and RUVBL2 polypeptides have been identified as subunits of several molecular machines that function in chromatin remodeling (e.g. INO80, NuA4), control transcription factor activity (e.g. cMyc, β-catenin), and help assemble complexes such as snoRNPs [42]. They have also been reported to interact with the phosphatidylinositol kinase-like kinases (PIKKs) ATM, ATR and DNA-PK, through which they can influence the efficiency of DNA damage response. In the studies published to date, the polypeptides were assumed to act together in heteromeric complexes ranging from heterodimers to heterododecamers [11,18]. In addition to creating a platform for assembly of the different multisubunit molecular machines, we now show that multimerization of RUVBL1/2 appears to be a stability requirement, as both polypeptides are degraded in the absence of their cognate partners (Fig 2A). Given the above evidence, our finding that the RUVBL1 and RUVBL2 signals separated during cytokinesis (Fig 1E) was unexpected. That the polypeptides could be detected in distinct subcellular structures indicates that they were protected from degradation even though they no longer interacted with one another. Lack of the cognate partner was shown to affect the stability of newly-synthesized populations of RUVBL1 and RUVBL2 rather than that of pre-existing complexes [34]. Our data suggest that RUVBL1 and/or RUVBL2 fate during mitosis is likely to be influenced by additional factors such as post-translational modifications, which might affect partner choice, degradation and compartmentalization of the polypeptides. Addressing these issues will require *ad hoc* studies focused exclusively on this phase of the cell cycle.

In an attempt to learn more about the role(s) of RUVBL1/2 in mitosis, we decided to knock down RUVBL1 expression by RNAi (Fig 2A). We observed a high incidence of lagging chromosomes during anaphase, which likely resulted from incorrect spindle attachments (Fig 2B and 2C). Defective spindle attachment would be expected to delay progression from mitotic entry to the onset of anaphase in RUVBL1-depleted cells, presumably by activation of the spindle assembly checkpoint. Yet, cells entered anaphase in the presence of unaligned chromosomes, which may be due to incomplete inhibition of the anaphase promoting complex/cyclosome by single unattached chromosomes [43,44], or because these unaligned chromosomes may be merotelically attached and thereby not detected by the spindle assembly checkpoint [44]. It is also possible that RUVBL1 depletion by RNAi impaired spindle assembly checkpoint signaling, as observed upon deregulation of other mitotic factors [45]. In agreement with our findings, a recent report [30] showed that RUVBL1/2 are required for chromatin decondensation at the end of mitosis in *Xenopus laevis* egg extracts and in human HeLa cells.

Co-localization of RUVBL1 and PLK1 at the intercellular bridge (Fig 4A), the evolutionary conservation of two PLK1 consensus sites in RUVBL1 (Fig 3A), the ability of recombinant PLK1 to modify T_239_ *in vitro* (Fig 3D) and the physical interaction between PLK1 and RUVBL1 during mitosis (Fig 4) strongly suggest that the kinase plays a role in the control of RUVBL1 function. PLK1 has been found to interact with RUVBL1/2 in a phospho-proteomic study of mitotic kinases [46] and it is tempting to speculate that this interaction may result in RUVBL1 phosphorylation on T_239_, which may enable it to dissociate from RUVBL2. Future experiments will show whether this is indeed the case.

The biological function of RUVBL1 and RUVBL2 remains enigmatic. Based on peptide sequence conservation, the polypeptides were predicted to be helicases. They possess the classical Walker A and B ATPase motifs, but their ATPase activity was not reproducibly observed [8–11,16]. In our hands, 3xFLAG-tagged RUVBL1 purified from transiently-transfected 293T cells displayed a robust ATPase activity, which was not stimulated by ssDNA. We therefore recommend that future experiments be carried out with polypeptides expressed in homologous systems.

The most informative way to study the biological roles of proteins is phenotypic analysis of cell lines lacking the polypeptides or expressing their variants. In the specific case of RUVBL1/2, downregulation of one polypeptide resulted in the degradation of the other, which made the study of phenotypes linked to the lack of only one of the subunits of this complex impossible. We therefore resorted to the use of “protein replacement” technology, whereby we induced the expression of FLAG-tagged murine variants (wild type or the ATPase-dead) in the human U2OS cell line, while concurrently expressing shRNA against endogenous RUVBL1, both under the control of doxycycline (Fig 5C). Because the murine variants were resistant to downregulation by the anti-human shRNA, and because RUVBL2 was not destabilized in this system, we were able to study selectively the effect of ablation of RUVBL1 ATPase. Doxycycline-dependent replacement of the endogenous human protein with murine wild type RuvBL1 had no detectable effect on the cells. In contrast, replacement of the wild type endogenous polypeptide with the murine ATPase-dead RUVBL1 brought about a dramatic growth defect, as measured in a colony formation assay (Fig 5F). Microscopic inspection of the RUVBL1 D_302_N-expressing cells showed no sign of programmed cell death and we therefore carried out flow cytometric analysis of the cell population, which showed most of the cells to be in the G1 phase of the cell cycle (Fig 5G), a result further confirmed by staining for cyclin A, a marker of S/G_2_ phase (Fig 5H). This analysis showed that the lack of RUVBL1 ATPase was insufficient to cause an arrest of the cells in mitosis, as already anticipated from the microscopic analysis of cell cycle progression in RUVBL1-knockdown cells (Fig 2B). However, the ATPase activity of RUVBL1 was essential for cell growth, even though it is impossible to say at this stage which of its numerous roles was responsible for this phenotype.

RUVBL1/2 is upregulated in a variety of cancers, including colon and liver [14,15,34,47] and the ATPase activity of RUVBL2 was shown to be required for sustained growth and viability of these tumor cells [48]. Our results show that RUVBL1 ATPase is essential for the growth of U2OS cells, which originate from an osteosarcoma. Should non-transformed cells be less dependent on RUVBL1, due to their intact checkpoints, small molecular weight inhibitors of RUVBL1 ATPase [49] might prove to be efficacious in the therapy of tumors that depend on this activity.

## Materials and Methods

### Cell culture and treatment

U2OS (human osteosarcoma), 293T (SV40 large-T antigen transformed human embryonic kidney cells) and Hela (human cervical carcinoma) cells were purchased from ATCC/LGC (Germany). U2OS T-REx were obtained from LifeTechnologies/Thermo Scientifc (Switzerland). Hela cells stably expressing GFP-tagged hRUVBL1 (Hela TDS), mRuvBL1, mRuvBL2 and ANLN (Hela Kyoto) were kindly provided by Ina Poser, MPI-Dresden. Cells were grown in D-MEM (Glucose/NaPyruvate), 10% FCS and Penicillin/Streptomycin and the respective selective antibiotics under 5% CO_2_ at 37°C. GFP-RUVBL1, GFP-RuvBL2 and GFP-ANLN expressing cells were grown in 400 μg/ml G418 (Geneticin, Invitrogen, 10131–019). For live microscopy, the cells were grown on LabTek chambered coverslips (Nunc). For mitotic arrest, cells were treated with 0.3 μg/ml nocodazole for 16 h and the loosely-attached cells were gently shaken off. The cell cycle profile was verified by flow cytometry. For double thymidine block and mitotic enrichment with nocodazole, cells were seeded 24 h prior treatment. Thymidine (1 mM) was added for 16 h, cells were released for 8 h and treated a second time with thymidine for 16 h. Five hours upon release from the second thymidine block, nocodazole (100 ng/ml) was added for 5 h.

### Immunofluorescence

Cells were seeded on cover slips and fixed with methanol for 15 min at -20°C or with 3.7% formaldehyde/PBS for 15 min at 4°C followed by permeabilization in 0.2% Triton X-100/PBS for 5 min at 4°C, and processed as previously described [50]. Images were taken on an Olympus IX81 fluorescence microscope, using a 60xOil/1.4/Ph objective (PlanApo, Olympus), and acquired with a CCD camera (Orca AG, Hamamatsu) using cell^R^ software (Olympus). The antibodies were anti-RUVBL1 (goat polyclonal sc-15259, Santa Cruz, 1:200), anti-RUVBL2 (rabbit polyclonal, generous gift of Irina Tsaneva, UCL London, 1:75), anti-T239 (rabbit polyclonal, custom-made by Eurogentec, 1:200), anti-PLK1 (mouse monoclonal P-5998, Sigma, 1:200), anti-FLAG (mouse monoclonal F-3165, Sigma, 1:1000) and anti-ß-Tubulin (mouse monoclonal T-4026, Sigma, 1:200), anti-Cyclin A (sc-596, *Santa Cruz*, 1:100) and anti-GFP (rabbit polyclonal Ab 290, *Abcam*, 1:1000). DNA was counterstained with DAPI.

### Confocal live imaging

Confocal live imaging was performed on a customized Zeiss LSM 510 Axiovert microscope using a 63x, 1.4 N.A. Oil Plan-Apochromat (Zeiss). The microscope was equipped with piezo focus drives (Piezosystem Jena), custom-designed filters (Chroma), and EMBL incubation chambers (European Molecular Biology Laboratory), providing a humidified atmosphere at 37°C with 5% CO_2_ throughout the experiment. Sample illumination was generally kept to a minimum and had no adverse effect on cell division and proliferation. Automated multi-location time-lapse movies and reflection-based autofocus on the LSM510 were controlled by in house-developed software based on macros as previously described [51]. Images were analysed with Zeiss LSM510 software.

### Immunoprecipitation

Cells were split 24 h after transfection and treated with nocodazole 48 h after transfection for 16 h, while one fraction was left untreated. Cells were lysed in NP-40 buffer (50 mM Tris-HCl pH 8.0, 150 mM NaCl, 1 mM EDTA, 1% NP-40, complete protease inhibitor (Roche), 1 mM NaF, 1 mM PMSF, 1 mM Na-vanadate) and 1 mg of protein extract was incubated with 3 μg anti-FLAG antibody or 30 μl supernatant of the 12CA5 hybridoma cell line producing anti-HA antibody. Immunoprecipitation was performed as previously described [52]. For GFP-tagged protein immunoprecipitation, 1 mg protein extract was incubated with 20 μl GFP-trap (Chromotek) and processed following the manufacturer’s protocol.

### siRNA transfections

HeLa cells were transfected (Oligofectine, Invitrogen) with siRNA oligo duplexes (MWG) for RUVBL1 (5’-AGA GCA UGU CGA AGA GAU Ctt-3’) or RUVBL2 (5’-GUC CGU GAG CAG AUC AAU Gtt-3’). Luciferase (GL2)-specific duplexes (MWG) served as a control. The cells were harvested after 48h, and mRNA- and protein levels were examined by RT-PCR and immunoblot analysis, respectively. For the inducible knockdown in U2OS cells, the RUVBL1 sequence listed above was cloned as shRNA into pSuperior (Addgene) and stably transfected.

### Western blot

Western blot analyses were performed as previously described [53]. Antibodies used were anti-RUVBL1 (sc-15259, *Santa Cruz*, 1:500), anti-RUVBL2 (gift from Matthias Gstaiger, ETH Zurich, 1:1000), anti-βTubulin (mouse monoclonal sc-5274, Santa Cruz, 1:1000), anti-TFIIH (rabbit polyclonal sc-293, Santa Cru*z*, 1:4000), anti-FLAG (F-3165, Sigma, 1:20000) and anti-GFP (mouse monoclonal, sc-9996, Santa Cruz, 1:500).

### Plasmid construction

Murine RUVBL1 and RUVBL2 were amplified from 18.81 cDNA with wt-fwd (5’-CCG GAA TTC ATG AAG ATT GAG GAG GTG AAG AG-3’) and wt-rev (5’-CCG CTC GAG TTA CTT CAT GTA CTT GTC CTG CTG-3’) oligos, cloned via *Eco*RI and *Xho*I in pcDNA3.1 (modified version) and expressed as an N-terminal HA-tagged fusion protein. The same construct was subcloned in pET28a(+) to obtain an N-terminal His-tagged expression vector. S_175_A, T_239_A and S_175_A/T_239_A mutants were generated using a standard *Quickchange* PCR-mutagenesis protocol. RUVBL2 was PCR-amplified from 18.81 cDNA using the oligos RUVBL2-fwd (5’-CCG GAA TTC ATG GCA ACC GTG GCA G-3’) and RUVBL2-rev (5’-CCG CTC GAG TCA GGA GGT GTC CAT TGT TTC-3’). The PCR product was digested with *Eco*RI and *Xho*I and cloned in two steps, since RUVBL2 contains an internal *Xho*I site, into pET28a(+) in order to express an N-terminal His-tagged fusion protein. For co-expression of GST-tagged RUVBL1 and His-tagged RUVBL2, RUVBL1 was subcloned into pGEX-2TK. All constructs were fully sequenced. The Flag-WT and D_302_N (ATPase-dead) mutant were cloned into pcDNA5/TO.

### Protein purification

Proteins were transiently expressed with an N-terminal 3xFLAG-tag in 293T cells, purified with anti-FLAG antibodies covalently bound to agarose beads, washed extensively with lysis buffer and eluted with 3xFLAG peptides. The eluates were dialyzed against 100 mM NaCl, 50 mM Tris-HCl, 10 mM MgCl_2_ and 1 mM DTT. Purification of proteins to near homogeneity was judged by silver staining.

Complex reconstitution for *in vitro* phosphorylation reactions was verified upon expression of His-RUVBL1/2 in *E*.*coli* and purification by Ni-NTA, Mono-Q and Superose 6 gel filtration chromatography.

FLAG-PLK1 was subcloned into pTXB3, expressed and purified using protocols previously described for Aurora-A [54].

### Kinase assay

The RUVBL1 mutants were incubated with purified PLK1 in the presence of [γ-^32^P]ATP (specific radioactivity, 5–10 βCi/nmol) for 15 min in a buffer containing 100 mM NaCl, 50 mM Tris-HCl, 10 mM MgCl_2_, 1 mM DTT. Samples were separated on a 7.5% or 10% SDS-PAGE and stained with Coomassie blue before exposure to a phosphor screen. Incorporation of radioactivity was detected with a Typhoon 9440 scanner and band intensities were quantified using ImageQuant5.2 software. To further analyze the phosphorylated peptides, the band corresponding to RUVBL1 was excised form the gel and digested with trypsin. The resulting peptides were spotted on a DC-cellulose plate and separated by hydrophobicity and charge [55].

### RT-PCR analysis

Cells were transfected with the indicated siRNA oligo duplexes 48 h before harvesting as described above. Total RNA was extracted with RNeasy-Kit (Qiagen). 500 ng of RNA were reverse-transcribed to cDNA by the use of reverse transcriptase and oligo(dT) primer according to standard protocols. For the following PCR reaction, one fiftieth of the obtained cDNA was used in combination with the following oligos: RUVBL1-fwd (5’-CTG TGT CAT CAG AGG CAC TGA-3’), RUVBL1-rev (5’-AAG TTC ACT GAT CTC TTC GAC ATG-3’); RUVBL2-fwd (5’-CAT CAC GCG AAT CCG G-3’), RUVBL2-rev (5’-TGA GTA GAC CCG CTT GAT GTC-3’); GAPDH-fwd (5’-CTC CTC TGA CTT CAA CAG CGA CAC-3’), GAPDH-rev (5’-CTC TCT CTT CCT CTT GTG CTC TTG C-3’).

### ATPase assay

The ATPase activity was assayed as previously described [56].

### Clonogenic survival assay

Clonogenic survival assay was performed as previously described [57]. Media were replaced every four days to ensure a constant doxycycline concentration (1 μg/ml). The assays were performed in triplicates.

### Flow cytometry

Propidium Iodide staining and flow cytometric analysis were performed as previously described [58].

## Supporting Information

S1 Fig Specificity of RUVBL1 staining. **(A)** Specificity of the RUVBL1 staining was ascertained by pre-incubating the antibody with recombinant His-RUVBL1 for 1h (His-RUVBL1:antibody, 10:1). Phase contrast images were taken as control. A merged image is shown with RUVBL1 (green) and DAPI (blue). **(B)** U2OS cells were transfected with RUVBL1 specific siRNA oligos 48 h prior fixation and staining with anti-RUVBL1 antibody. DNA is counterstained with DAPI (blue). **(C)** A pattern similar to that observed in A was obtained using a different anti-RUVBL1 antibody.
(PDF)

S2 Fig RUVBL1 depletion gives rise to lagging chromosomes. U2OS T-REx cells stably-transfected with a doxycycline-inducible shRNA against endogenous RUVBL1 were co-transfected with a doxycycline-inducible shRNA-resistant FLAG-tagged murine RuvBL1 construct and treated or not with doxycycline for 48 h, as indicated. Protein expression was verified by immunoblotting (**A**) and occurrence of lagging chromosomes was quantified by analyzing >75 anaphases for each cell line and condition **(B)**.
(PDF)

S3 Fig Sequence alignment of RUVB-like proteins. **(A)** Protein sequences from human RUVBL1 (NP_003698) and RUVBL2 (NP_006657) were obtained from http://www.ncbi.nlm.nih.gov and aligned with http://www.ncbi.nlm.nih.gov/blast/bl2seq/wblast2.cgi using default parameters. Alignment was processed using Boxshade 3.2, with identical amino acids in black and homologous amino acids in gray boxes. The sequence was colored according to the domain structure, with domain 1 in orange, domain 2 in blue and domain 3 in red, respectively. Walker A and Walker B motifs are highlighted with black rectangles and potential PLK1 phosphorylation motifs with red rectangles, respectively. **(B)** Sequence comparison of human RUVBL1 with RuvB of *Thermotoga maritima* (AAB03727). **(C)** The structure of RUVBL1 is shown with domains highlighted in the colors used above. Threonine at position 239 in RUVBL1 is highlighted in turquoise. The structure was modified based on published data [10] using PyMOL software and the PBD files 2c9o (for RUVBL1) and 1in7 (for RuvB), respectively.
(PDF)

S4 Fig *In vitro* phosphorylation of RUVBL1 by PLK1. **(A)** Different amounts of purified His-tagged RUVBL1 were incubated with PLK1 in the presence of [γ-^32^P]ATP. Casein served as positive control. Proteins were separated by SDS-PAGE and the Coomassie blue-stained gel was subjected to autoradiography. **(B)** His-tagged RUVBL1 mutants were purified to near homogeneity and subjected to SDS-PAGE and Coomassie blue staining. **(C)** RUVBL1 can be phosphorylated while in complex with RUVBL2. GST-tagged RUVBL1 and His-tagged RUVBL2 were co-expressed in *E*. *coli* and purified using GSH beads. Co-purification of RUVBL2 confirmed complex formation, which was further assessed by size exclusion chromatography (data not shown). GST-RUVBL1 and GST alone served as controls in the kinase reaction.
(PDF)

S5 Fig Cells expressing an ATPase-dead RuvBL1 fail to proliferate. Colony survival assay monitoring long-term survival after induction of wild type or ATPase-dead FLAG-tagged murine RuvBL1 and simultaneous down-regulation of endogenous human RUVBL1. Cells were seeded in low density and colonies were stained and counted 14 days later. Assays were carried out in triplicates and numbers were normalized against untreated cells.
(PDF)
