# Supplementary figures and images for: Chromosome Missegregation Associated with RUVBL1 Deficiency
Source: PLoS One. 2015 Jul 22;10(7):e0133576. doi: 10.1371/journal.pone.0133576 (PMC4511761; doi:10.1371/journal.pone.0133576)

Figure S1

Gentili et al.

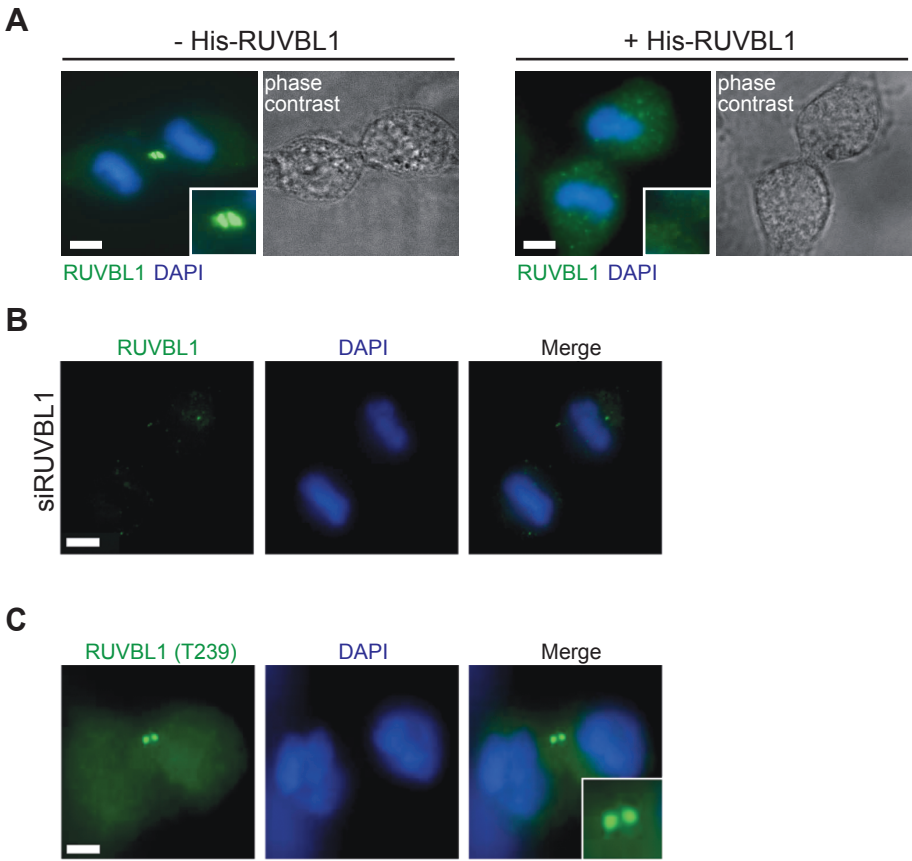

Supplement: S1 Fig — (A) Specificity of the RUVBL1 staining was ascertained by pre-incubating the antibody with recombinant His-RUVBL1 for 1h (His-RUVBL1:antibody, 10:1). Phase contrast images were taken as control. A merged image is shown with RUVBL1 (green) and DAPI (blue). (B) U2OS cells were transfected with RUVBL1 specific siRNA oligos 48 h prior fixation and staining with anti-RUVBL1 antibody. DNA is counterstained with DAPI (blue). (C) A pattern similar to that observed in A was obtained using a different anti-RUVBL1 antibody. (PDF) [file pone.0133576.s001.pdf]

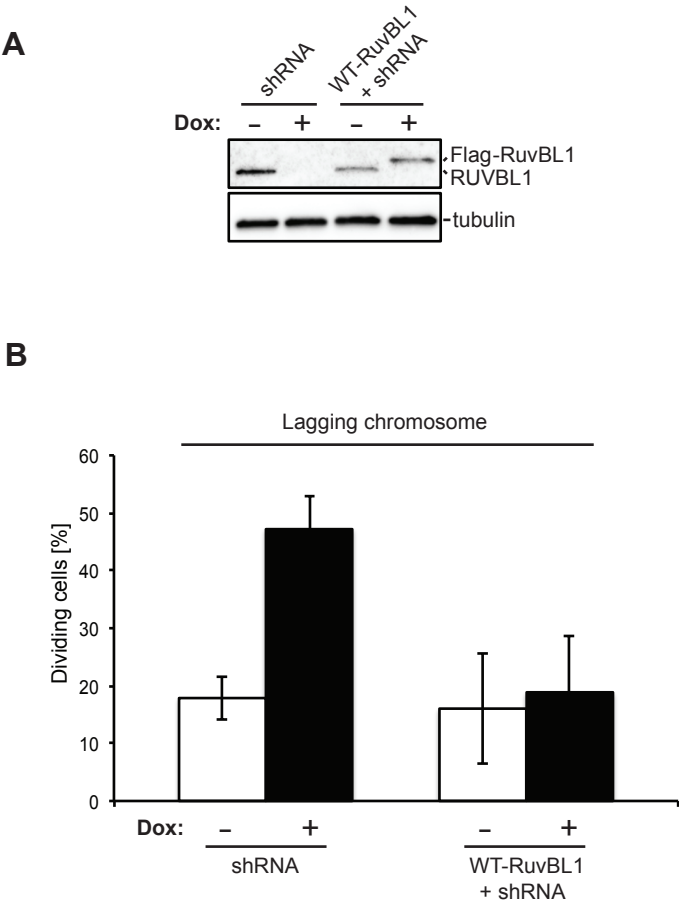

Supplement: S2 Fig — U2OS T-REx cells stably-transfected with a doxycycline-inducible shRNA against endogenous RUVBL1 were co-transfected with a doxycycline-inducible shRNA-resistant FLAG-tagged murine RuvBL1 construct and treated or not with doxycycline for 48 h, as indicated. Protein expression was verified by immunoblotting (A) and occurrence of lagging chromosomes was quantified by analyzing >75 anaphases for each cell line and condition (B). (PDF) [file pone.0133576.s002.pdf]

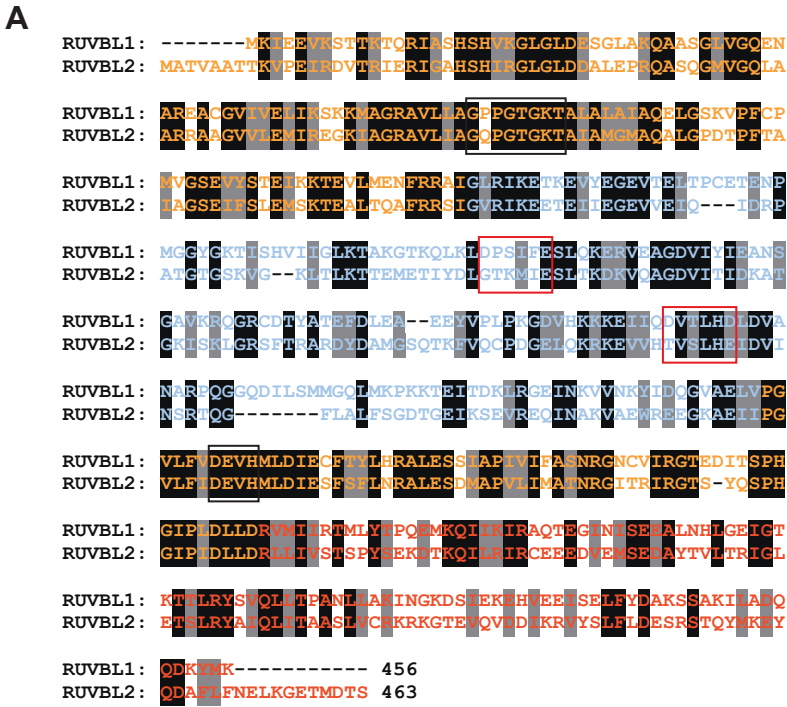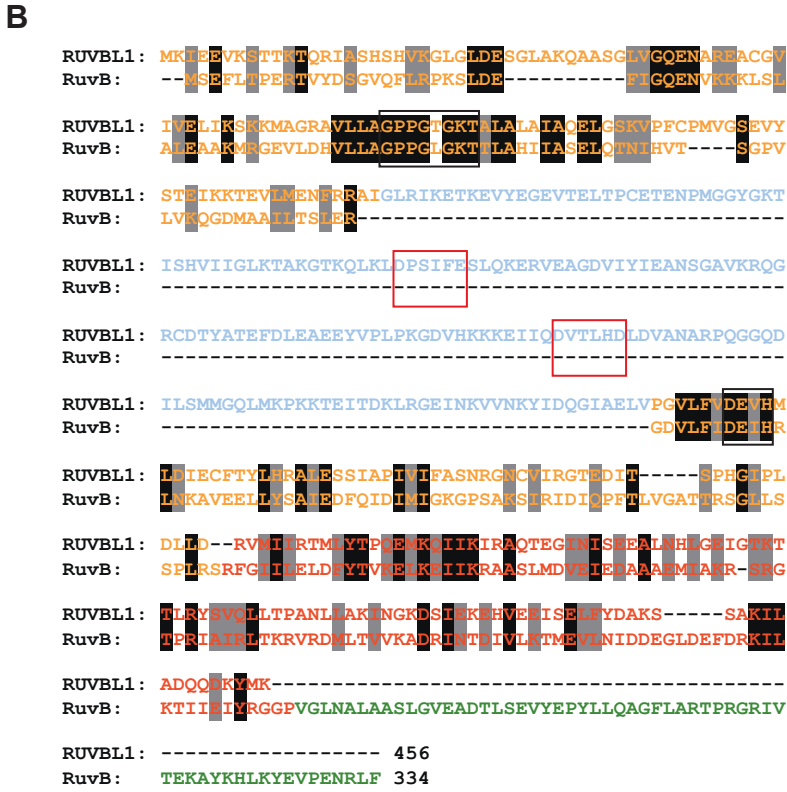

**C** RUVBL1 (*Homo sapiens*)    RuvB (*Thermotoga maritima*)

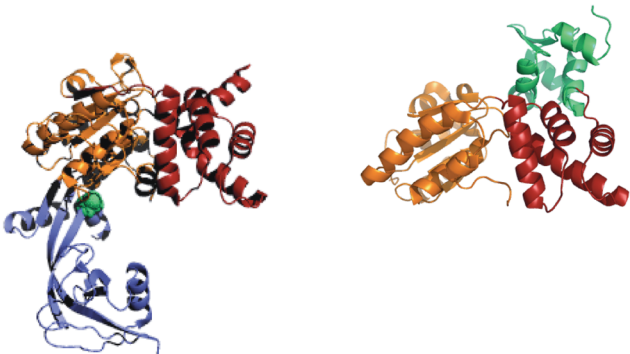

Supplement: S3 Fig — (A) Protein sequences from human RUVBL1 (NP_003698) and RUVBL2 (NP_006657) were obtained from http://www.ncbi.nlm.nih.gov and aligned with http://www.ncbi.nlm.nih.gov/blast/bl2seq/wblast2.cgi using default parameters. Alignment was processed using Boxshade 3.2, with identical amino acids in black and homologous amino acids in gray boxes. The sequence was colored according to the domain structure, with domain 1 in orange, domain 2 in blue and domain 3 in red, respectively. Walker A and Walker B motifs are highlighted with black rectangles and potential PLK1 phosphorylation motifs with red rectangles, respectively. (B) Sequence comparison of human RUVBL1 with RuvB of Thermotoga maritima (AAB03727). (C) The structure of RUVBL1 is shown with domains highlighted in the colors used above. Threonine at position 239 in RUVBL1 is highlighted in turquoise. The structure was modified based on published data [10] using PyMOL software and the PBD files 2c9o (for RUVBL1) and 1in7 (for RuvB), respectively. (PDF) [file pone.0133576.s003.pdf]

Figure S4 Gentili et al.

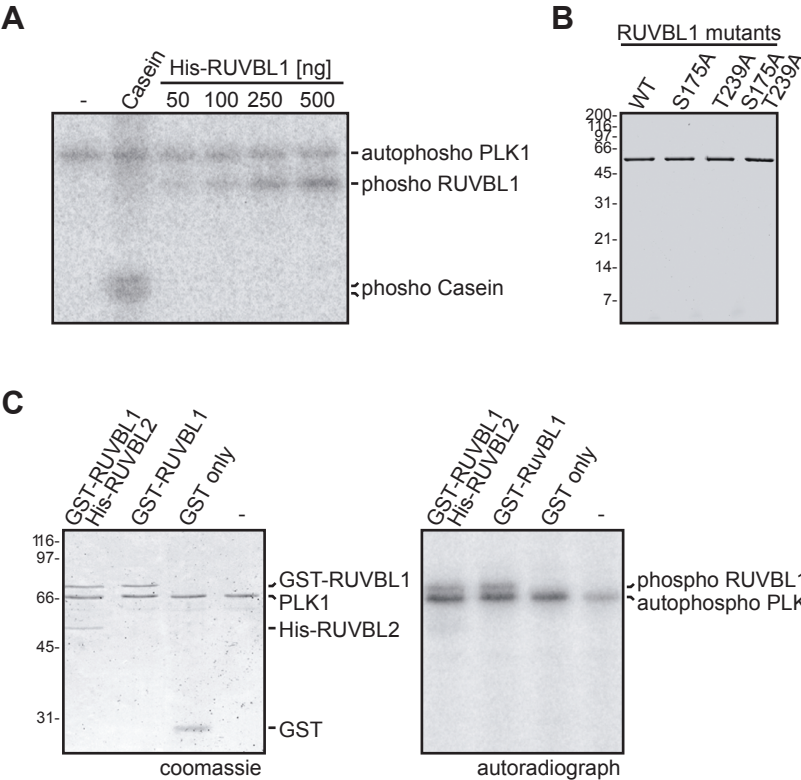

Supplement: S4 Fig — (A) Different amounts of purified His-tagged RUVBL1 were incubated with PLK1 in the presence of [γ-32P]ATP. Casein served as positive control. Proteins were separated by SDS-PAGE and the Coomassie blue-stained gel was subjected to autoradiography. (B) His-tagged RUVBL1 mutants were purified to near homogeneity and subjected to SDS-PAGE and Coomassie blue staining. (C) RUVBL1 can be phosphorylated while in complex with RUVBL2. GST-tagged RUVBL1 and His-tagged RUVBL2 were co-expressed in E. coli and purified using GSH beads. Co-purification of RUVBL2 confirmed complex formation, which was further assessed by size exclusion chromatography (data not shown). GST-RUVBL1 and GST alone served as controls in the kinase reaction. (PDF) [file pone.0133576.s004.pdf]

**Figure S5**      **Gentili et al.**

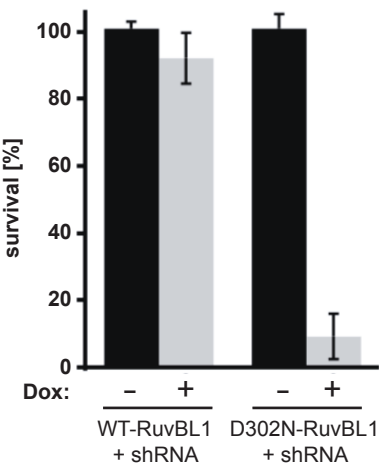

Supplement: S5 Fig — Colony survival assay monitoring long-term survival after induction of wild type or ATPase-dead FLAG-tagged murine RuvBL1 and simultaneous down-regulation of endogenous human RUVBL1. Cells were seeded in low density and colonies were stained and counted 14 days later. Assays were carried out in triplicates and numbers were normalized against untreated cells. (PDF) [file pone.0133576.s005.pdf]
